# Population Substructure and Control Selection in Genome-Wide Association Studies

**DOI:** 10.1371/journal.pone.0002551

**Published:** 2008-07-02

**Authors:** Kai Yu, Zhaoming Wang, Qizhai Li, Sholom Wacholder, David J. Hunter, Robert N. Hoover, Stephen Chanock, Gilles Thomas

**Affiliations:** 1 Division of Cancer Epidemiology and Genetics, National Cancer Institute, Rockville, Maryland, United States of America; 2 Core Genotyping Facility, Advanced Technology Program, SAIC Frederick, Inc. National Cancer Institute - Frederick, Frederick, Maryland, United States of America; 3 Academy of Mathematics and Systems Science, Chinese Academy of Sciences (CAS), Beijing, China; 4 Program in Molecular and Genetic Epidemiology, Department of Epidemiology, Harvard School of Public Health, Boston, Massachusetts, United States of America; Copenhagen University Hospital, Denmark

## Abstract

Determination of the relevance of both demanding classical epidemiologic criteria for control selection and robust handling of population stratification (PS) represents a major challenge in the design and analysis of genome-wide association studies (GWAS). Empirical data from two GWAS in European Americans of the Cancer Genetic Markers of Susceptibility (CGEMS) project were used to evaluate the impact of PS in studies with different control selection strategies. In each of the two original case-control studies nested in corresponding prospective cohorts, a minor confounding effect due to PS (inflation factor *λ* of 1.025 and 1.005) was observed. In contrast, when the control groups were exchanged to mimic a cost-effective but theoretically less desirable control selection strategy, the confounding effects were larger (*λ* of 1.090 and 1.062). A panel of 12,898 autosomal SNPs common to both the Illumina and Affymetrix commercial platforms and with low local background linkage disequilibrium (pair-wise *r* ^2^<0.004) was selected to infer population substructure with principal component analysis. A novel permutation procedure was developed for the correction of PS that identified a smaller set of principal components and achieved a better control of type I error (to *λ* of 1.032 and 1.006, respectively) than currently used methods. The overlap between sets of SNPs in the bottom 5% of p-values based on the new test and the test without PS correction was about 80%, with the majority of discordant SNPs having both ranks close to the threshold. Thus, for the CGEMS GWAS of prostate and breast cancer conducted in European Americans, PS does not appear to be a major problem in well-designed studies. A study using suboptimal controls can have acceptable type I error when an effective strategy for the correction of PS is employed.

## Introduction

Genome-wide association studies (GWAS) have emerged as an effective approach to identify common polymorphisms underlying complex traits [1]–[4]. In place of a family-based design frequently employed in linkage scans, GWAS use a case-control design primarily because of its efficiency in investigating a large number of common variants in the genome together with the availability of sufficiently large collections of unrelated cases with or without coordinated collections of controls.

The presence of population stratification (PS)—allele frequency differences between cases and controls due to systematic ancestry differences—can lead to greater than nominal type I error rate [5]–[11]. Differences in the origin of populations of cases and controls can arise if the two groups are recruited independently or have different inclusion criteria. Differences in ancestry between cases and controls can also occur even if cases and controls are drawn from the same heterogeneous population, such as the European American population, when the disease risk varies across subpopulations due to differences in distribution of unmeasured risk factors [5]. Although the potential for an increase in false positives in well-designed association studies conducted in a stratified population is indisputable 6, 7, 12, the extent and impact of PS on case-control association studies in practice, particularly in GWAS, can now be thoroughly investigated as empirical evidence from recent association studies becomes available.

One principle of classical epidemiologic study design is that the distribution of risk factors of interest in controls should be the same as the distribution in the population from which cases have been ascertained [13]. A population-based study satisfies this principle by choosing a random sample of controls from the same population from which cases are selected. Violation of this principle in the studies of genetic effects may be of less concern than in the studies of environmental risk factors, if the distribution of population ancestry in individual cases and controls is available and can be used to control the type I error rate at the cost of only a modest drop in power. Principal component analysis [14]–[17] or other methods [18], [19] can be used to estimate the population ancestry from the genotypes on a panel of SNPs not associated with the disease status. The SNP panel can be selected from the large number of SNPs typed in GWAS, of which the vast majority (>99%) are not expected to be related to the disease under study. Furthermore, a second set of SNPs, minimally correlated with the previous one chosen for ancestry inference can be used to evaluate the extent of confounding by PS as well as the effectiveness of the correction for PS, by comparing the distribution of the test statistic (with or without the correction for PS) observed over the second set of SNPs with its expected distribution under the null hypothesis. Systematic inflation in the observed statistics would indicate that ancestry effects have not been fully controlled. Thus, we postulate that the analysis of thousands of well-chosen SNPs distributed throughout the genome could permit relaxation of the requirement that cases and controls share the same population of origin. In this regard, it is possible to analyze cases and controls recruited from independently designed studies or allow the use of a single, common control group for a variety of disease groups, a strategy successfully used by the Welcome Trust Case Control Consortium (WTCCC) to identify novel variants in a number of common diseases [20].

The Cancer Genetic Markers of Susceptibility (CGEMS) project has conducted two multi-stage GWAS in breast cancer and prostate cancer [1], [3], [21]. For each disease, the initial genome-wide scan was performed in a nested case-control study drawn from a prospective cohort in self-described European Americans. The CGEMS data provides the opportunity for empirical evaluation of the impact of population stratification in an optimal study design. By exchanging the control groups of the two studies, we have explored the consequences of the non-standard strategy of using external controls. We thus can make comparisons between the two approaches.

We identified a set of autosomal SNPs common to both the Illumina and Affymetrix commercial platforms that can robustly monitor residual population structure in European American populations. One commonly used approach for the correction of PS is to adjust simultaneously for a fixed number of top-ranked principal components (PCs) resulting from a principal component analysis [15], [16]. However, this approach may have an overly negative impact on the power if the cases and controls are equally distributed along the selected PCs, or if the adjustment of certain covariates (such as self identified ethnicity, or recruitment center) already included in the association analyses correctly maps to major axes of genetic heterogeneity. To efficiently identify the relevant PCs and keep their number to a minimum while allowing an effective correction, we have developed a permutation procedure to evaluate their effectiveness on PS correction as additional PCs are taken for adjustment in the association test. Taken together, these developments provide a procedure that should be helpful for both PS evaluation and adjustment in GWAS.

## Materials and Methods

### Study material

Both the genome-wide scans used in this study analyzed approximately 550,000 SNPs on the Illumina platform. The prostate cancer study genotyped cases and matched controls collected from the Prostate, Lung, Colorectal and Ovarian (PLCO) Cancer Screening Trial using the HumanHap300 (Illumina, San Diego, CA) and HumanHap240 chips (Illumina, San Diego, CA) [1]. The breast cancer scan used the HumanHap550 chip (Illumina, San Diego, CA), which is equivalent to the HumanHap300 and HumanHap240 chips combined, to genotype cases and their matched controls collected from the Nurses' Health Study (NHS) cohort [2]. In both studies, participants were restricted to individuals who were of self-described European descent. Quality control and quality assessment removed subjects with low completion rates (<90%), subjects with evidence of an intercontinental admixture (European admixture coefficient less than 90%, estimated by STRUCTURE [19]), and removal of one of each pair of first-degree relatives (identified using PREST [22]). No second degree relatives were detected. For this study, the test set for the PLCO prostate cancer study consisted of 1,171 prostate cancer cases and 1,094 controls while the test set for the NHS breast cancer study included 1,140 breast cancer cases and 1,138 controls. Four combinations of cases and controls were analyzed: PLCO cases vs. PLCO controls (PLCOca-PLCOco), NHS cases vs. NHS controls (NHSca-NHSco), PLCO cases vs. NHS controls (PLCOca-NHSco), and NHS cases vs. PLCO controls (NHSca-PLCOco).

Further data cleaning of the autosomal SNPs typed in both PLCO and NHS scans retained SNPs with MAF >5%, a P-value for fitness for Hardy-Weinberg proportion equilibrium exact test >10^−5^ in both control sets, and a rate of missing genotypes <5%. A handful of SNPs that had different genotype frequencies between the PLCO controls and NHS controls (with P-value <10^−7^ based on the 2-df chi-squared test) were removed, most likely due to informatic inconsistencies in SNP identification between studies. In total, 475,116 autosomal SNPs (hereafter called the testing SNPs) were identified for further analysis.

### Algorithm to select a set of SNPs for population structure inference

To optimize the principal components analysis of population structure, we identified a set of SNPs with low background LD, i.e., *r* ^2^ LD statistic [23] less than a given threshold 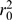 (e.g., 0.004) within a given physical distance *d* (e.g., 500 kb). Our algorithm modifies the greedy search algorithm of Carlson et al. [24], which selects the minimum number of SNPs (called tagSNPs) necessary to monitor remaining non-tagSNP above a threshold level of correlation (measured by *r* ^2^). For our purposes, the SNP selection algorithm differs in that it identifies the maximum number of mutually “independent” SNPs for the inference of population structure.

The algorithm selected a panel of population structure inference SNPs by iterating over the following three steps. First, for each SNP (called the reference SNP in this process) in the selection pool, all SNPs that are within the distance *d* of the reference SNP and have the *r* ^2^ LD measure with the reference SNP above the threshold 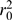 were identified and grouped as a bin. Second, the bin with the smallest size is identified, with its reference SNP being added to the list of structure inference SNPs. If more than two bins have the minimal size, we randomly pick one. Third, the selection pool of SNPs is updated by removing every SNP included in the bin identified in the second step. The process is complete when no SNP is left in the selection pool.

The above algorithm can be used with no prior information concerning population structure but when prior information on the population structure, such as ethnic background, is available, it may be of interest to identify a smaller set of highly informative SNPs. In this case, the criteria suggested by Pfaff et al [25] can be used in the bin selection applied in step 2. Also the algorithm can be modified in a straightforward manner to expand an existing list of structure inference SNPs from an augmented pool of SNPs.

### Statistical analysis

Here we provide more details on main statistical methods used in the analyses.

#### Principal component analysis

We chose a panel of *M* structure inference SNPs for the detection and correction of population substructure in a GWAS with a total of *N* cases and controls. The genotype at a marker locus is coded as 0, 1 or 2, corresponding to the copy number of an arbitrary allele. Let *g_i,m_* be the genotype measured at SNP *m* for the *i*th subject, 1≤*i*≤*N*, 1≤*m*≤*M*. The PCA summarizes the information measured on *M* structure inference SNPs and represents study participants by their projected positions (called principal components, or PCs) along a few orthogonal axes with “large” genetic variations.

There are various forms of PCA for genetic data depending on how the covariance matrix is calculated. Here we follow the EIGENSTRAT method [15], [16]. We first standardize each genotype coding as 
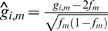
, with 
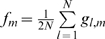
 being the allele frequency for the *m* ^th^ marker. In the following discussion, we always use the standardized genotype and still represent it as *g_i,m_*, and organize all genotypes into the matrix **G** = (*g_i,m_*)*_N_*_×*M*_. We obtain the sample covariance matrix 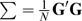 for the *M* markers. For the PCA, we find the first *L* (say *L* = 3) largest eigenvalues of Σ, and their corresponding normalized eigenvectors, **v**_1_, **v**_2_, …, **v** *_L_*, with **v** *_l_* = (*v_l_*_,1_,*v_l_*_,2_,…,*v_l_*_,*M*_)′, 1≤*l*≤*L*. For the *i*th subject with genotypes (standardized as above) **g** *_i_* = (*g_i_*_,1_,*g_i_*_,2_,…,*g_i_*_,*M*_)′, its *l*th principal component (PC) is given by 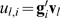, 1≤*l*≤*L*. Thus, **v** *_l_* defines the PC direction with the *l*th largest “genetic” variation and *u_l,i_* is the *i*th subject's projected position onto this axis. Following Patterson et al., the significance level of the genetic variation along a given PC direction is evaluated by the Tracy-Widom test [16].

In the eigenvector **v** *_l_* = (*v_l_*_,1_,*v_l_*_,2_,…,*v_l_*_,*M*_)′, its *m*th element *v_l,m_*, called loading, 1≤*m*≤*M*, reflects the influence of *m*th marker on shaping **v** *_l_*. Following suggestions from the WTCCC, the loading of every SNP on the **v** *_l_* can be used to search for chromosomal regions where variation pattern among samples would determine the *l*th PC direction. A PC that reflects regional genetic variation will be evidenced by a spike of high loadings for markers in that chromosomal region.

#### Measuring the correlation between two PC directions

If two groups of subjects (e.g., PLCOca-PLCOco and NHSca-NHSco) are collected from the same population, we expect the significant PC directions from both groups to point in similar directions. We can quantify the similarity of two directions using the Spearman rank correlation coefficient of the loading of every SNP along these directions.

#### Between-groups comparison along a PC direction

The confounding effect of PS only occurs when systematic population structure differences exist between cases and controls. Once one or more PC directions with large genetic variation are identified through PCA, comparison of PCs for cases along that axis with those of controls using the Wilcoxon rank sum test can be informative. This is a non-parametric test for evaluating whether two sets of observations have comparable distribution.

#### Selection of PC for PS correction

An approach commonly used to correct for PS is to adjust simultaneously for a fixed number of top- ranked PCs or for those PCs that have significantly large genetic variation according to the Tracy-Widom test [15], [16]. This approach might not be optimal if selected PCs are distributed almost equally in cases and controls [26]. In Text S1, we have presented an illustrative example of how unnecessary adjustment of population substructure (even one PC) could lead to a significant loss in power (Text S1, Table S1).

Our selection procedure attempts to include only PCs that are useful for the correction of PS. A PC is chosen whenever its inclusion can significantly reduce the inflation in type I error rate, measured by the over-dispersion factor, in comparison with the addition of a randomly generated covariate. Once a set of PCs has been identified, they are adjusted with other covariates in a standard logistic regression model.

To apply this procedure, it is necessary to determine a set of markers for which the inflation in type I error for the chosen test statistic (with or without PC adjustment) can be properly measured. Ideally, to avoid bias, this set of markers should be uncorrelated with the set of markers used for PC detection, and not associated with the disease risk. To such an end, we can identify a large set **S** (approximately 240,000 SNPs) of genomic control markers that do not exceed a threshold level of LD with any SNPs used in the PCA. Although several disease-related SNPs might be included in this set, their effect on the inflation estimation can be ignored as the vast majority of the SNPs in the set **S** would not be disease-related. For each genomic control SNP in **S**, assuming an additive genetic model, a 1-df Wald test can be performed by adjusting any chosen PCs and other covariates. Following Devlin and Roeder [27], the over-dispersion factor can be estimated as the median of Wald test statistics over the set of genomic control SNPs divided by the expected median of the chosen test statistics under the null hypothesis (i.e., in the present case 0.456). The intent in our study is to use the over-dispersion factor for the purpose of evaluating the effectiveness in the correction of PS by adjusting for a given set of PCs, but not for the adjustment for association testing results as originally proposed by others [27], [28]. The over-dispersion factor provides a summary measure for the inflation level in the type I error. Although, as we show below, the over-dispersion factor tends to be positively correlated with empirical type I errors under various considered significant levels, there is no simple analytic relationship between them. For example, in our analysis of NHSca-PLCOco described later, the over-dispersion factor at the level of 1.06 corresponds to 14%, 20% and 30% inflation in type I error under the significant level of 0.05, 0.01 and 0.001, respectively.

To limit the searching space, we focus on only PCs with significantly large genetic variation (with P-values less than 0.05 based on the Tracy-Widom test). The number of significant PCs, represented by *L*, is either 4 or 5 among the four considered scans (the two original and the two reconstructed). Let **u** *_l_* = (*u_l_*_,,1_,*u_l_*_,2_,…,*u_l_*_,*N*_)′, 1≤*l*≤*L* be the vector of the *l*th PCs for all subjects. To reduce the computing time further, we order those *L* PCs **u**_1_, **u**_2_, …, **u** *_L_* according to their Wilcoxon rank-sum test statistics that compare the distributions between cases and controls along individual PCs, and define them in that order as **u**_(1)_, **u**_(2)_, …, **u**_(*L*)_, with **u**_(1)_ being the PC with the largest Wilcoxon rank-sum test statistic. We use the following greedy search algorithm to choose a subset of PCs for the correction of PS by sequentially evaluating **u**_(1)_, **u**_(2)_, …, **u**_(*L*)_.

1. Define **E** to be the set of selected PCs, starting with **E** = *φ*, the empty set.
2. Iterate the following two steps for *l* = 1 to *L*.
  - Use a permutation procedure (described below) to evaluate the empirical P-value associated with the ‘impact” of adjusting for the PC **u**_(*l*)_ in addition to those already in **E**.
  - If the estimated P-value is less than a chosen threshold (say, 0.05), expand the set **E** by adding **u**_(*l*)_, otherwise, leave the set **E** unchanged
3. The set **E** at the end of *L* iterations is the final set of PCs to be adjusted in the association test.

The permutation procedure used in step 2.a is designed to determine if adjusting for the PC **u**_(*l*)_ in addition to those already included in **E** results into a significantly lower over-dispersion factor, compared with the level expected under the situation when the additional covariate to be adjusted for is randomly generated, and thus has no contribution to the correction of PS. The rationale is that we should adjust for **u**_(*l*)_ whenever its inclusion makes a “significant” impact on the correction of PS. Here are the basic steps for the permutation procedure used in the *l*th iteration of the search algorithm.

- Obtain the “observed” over-dispersion factor 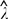 by applying the test with adjustment for the PC **u**_(*l*)_ as well as those already included in **E** on SNPs from the set **S**.
- Randomly permute entries in the vector **u**_(*l*)_ a predetermined number of times, *B*, and denote the resulting permuted version of **u**_(*l*)_ as 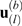, *b* = 1,…,*B*.
- Based on 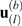, *b* = 1,…,*B*, obtain the over-dispersion factor *λ* ^(*b*)^ by applying the test with adjustment for **u** ^(*b*)^ as well as those included in **E** on SNPs within the set **S**.
- The empirical P-value associated with the PC **u** ^(*b*)^ is estimated as the proportion of *λ* ^(*b*)^, *b* = 1,…,*B*, that are smaller than the “observed” 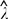.

In the above permutation procedure, each *λ* ^(*b*)^ is the over-dispersion factor based on the test adjusting for covariates included in **E** as well as 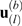, which is a the permuted version of **u**_(*l*)_. All *λ* ^(*b*)^, *b* = 1,…,*B*, provide the reference distribution for the evaluation of 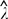. We find in our numerical experiments that the median of *λ* ^(*b*)^, *b* = 1,…,*B*, is roughly at the same level as *λ*_Current_, the over-dispersion factor for the test adjusting for only covariates included in **E**. Thus, if the additional adjustment of **u**_(*l*)_ (in addition to the ones already in **E**) does not lead to an over-dispersion factor 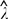 that is lower than the current level (*λ*_Current_), we skip the permutation procedure and do not choose **u**_(*l*)_.

In our analyses, we used 100 permutation steps and approximately 240,000 genomic control SNPs for the evaluation of the over-dispersion factor in each permutation step. Thus, the permutation procedure may be computationally intensive. If necessary, the computing time may be shortened by selecting SNPs that are unrelated (i.e., low background LD).

#### Implementation

The algorithm for the selection of structural inference SNPs was implemented in the Python programming language. All the other analyses were conducted using the open-source R language.

## Results

### The set of structure inference SNPs

To assemble a set of common SNPs informative for inference of population substructure (called structure inference SNPs) for GWAS, initially we identified a set of 40,817 autosomal SNPs common to Affymetrix 500 k, Illumnia HumanHap300 and Illumina HumanHap550, filtered on the basis of a completion rate greater than 95% in both CGEMS scans, minor allele frequency (MAF) >5%, and a fitness for Hardy-Weinberg proportion exact test P-value >10^−3^ in both control sets. From this pool of SNPs, using our described selection algorithm we selected 12,898 structure inference SNPs that had low background LD in the joint PLCO and NHS control samples (*r* ^2^ less than 0.004 for any pair located within 500 kb on the same chromosome). The detailed list is provided in the Table S2, together with a visual representation of the position and observed MAF of the SNPs on the chromosomes (Figure S1).

The set of structure inference SNPs was used in the PCA to detect axes with large genetic variations for the two original genome wide scans and the two test studies in which controls were swapped between PLCO and NHS. Inspection of the SNP loadings plotted along each chromosome indicated that none of the top 5 PCs from each of 4 studies showed evidence of being driven by regional genetic variation pattern (see example of Figure S2).

Using a slightly modified version of the SNP selection algorithm described in the Material and Methods Section, from the list of 475,116 testing SNPs monitored by the Illumina HumanHap550 chip, we identified a new second set of 7,017 structure inference SNPs that are locally uncorrelated (*r* ^2^ less than 0.004 for any pair located less than 500 kb apart on the same chromosome) among themselves and similarly uncorrelated to the 12,898 SNPs already identified. The dependence of the PCA conclusions on the set of structure inference SNPs can be evaluated by comparing the results using the first set of 12,898 SNPs to those obtained with the second set of 7,017 SNPs

### The set of genomic control SNPs for the assessment of over-dispersion

**Figure 1:**
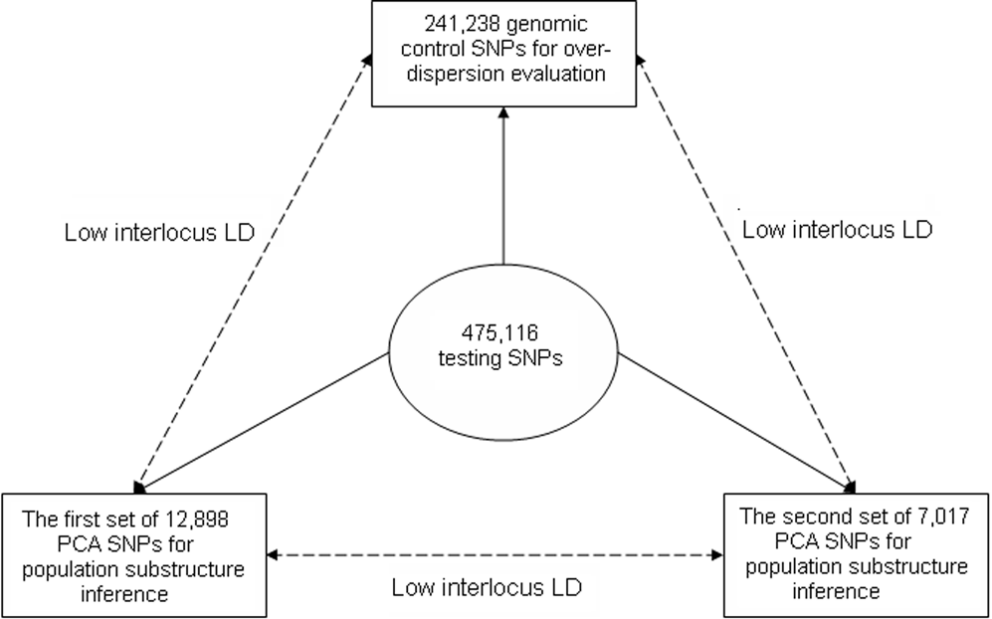
A diagram for the three main sets of SNPs used in the text. The first set of PCA SNPs is used to identify hidden population substructure. The set of genomic control SNPs is used to evaluate the over-dispersion factor in a given study, as well as in the proposed permutation procedure to select relevant PCs for the correction of PS. The second set of PCA SNPs is used to validate findings from the first set of PCA SNPs. In applications, only the first set of PCA SNPs is recommended.

For association tests without PC adjustment, an unbiased evaluation of the over-dispersion factor *λ* can be obtained using all the 475,116 testing autosomal SNPs. For evaluation after PC adjustment, it is however important to avoid the potential bias that can arise from SNPs highly correlated to the initial structure inference SNP set. Thus, *λ* is better estimated based on testing statistics measured on a set of genomic control SNPs with no correlation with the set of structure inference SNPs. To remain consistent throughout the analyses, the over-dispersion factor *λ* for tests with or without PC adjustment was systematically evaluated with the same set of 241,238 genomic control (autosomal) SNPs having r^2^ less than 0.01 with any nearby (within a distance of 500 kb) structure inference SNP from either the 12,898 or the 7,017 SNP sets. See Figure 1 for a summary of the relationship among the three sets of SNPs.

### Population structure in the PLCO prostate cancer study

We applied the PCA using the set of 12,898 structure inference SNPs in the original nested case-control study of prostate cancer (PLCOca-PLCOco) and found that the top 4 PCs are strongly significant with P-values less than 10^−4^ based on the Tracy Widom test, while the 5^th^ is borderline significant (Table 1). To further justify the existence of axes with large genetic variation, we conducted a new PCA on PLCOca-PLCOcn using the alternative 7,017 structure inference SNPs described above (Table S3). In this case, the first two PCs were highly significant, namely a Tracy-Widom test P-value <10^−7^, but the additional lower ranked PCs (third and onwards) had P-value larger than 0.05. It is notable that there is a significant correlation for the first, as well as the second PC between the two PCAs (with Spearman rank correlation coefficient larger than 0.5 and P-value less than 10^−15^). Since the lower ranked (third and onwards) PCs estimated by the smaller set of SNPs were not significant, their correlations with the ones estimated by the larger set of SNPs were not evaluated.

**Table 1:** Tracy-Widom tests and associated P-values (in parenthesis) for the significance of principal components.

	PLCOca-PLCOco^a^	PLCOca-NHSco^b^	NHSca-NHSco^c^	NHSca-PLCOco^d^
PC #1	514.40 (<10^−7^)	572.44 (<10^−7^)	771.37 (<10^−7^)	722.58 (<10^−7^)
PC #2	179.90 (<10^−7^)	211.20 (<10^−7^)	160.31 (<10^−7^)	142.95 (<10^−7^)
PC #3	9.90 (<10^−7^)	9.63 (<10^−7^)	35.38 (<10^−7^)	27.69 (<10^−7^)
PC #4	4.52 (7.0×10^−5^)	1.05 (0.045)	9.66 (<10^−7^)	3.54 (5.8×10^−4^)
PC #5	1.02 (0.047)	−0.34 (0.238)	−0.16 (0.168)	2.07 (9.2×10^−3^)

Note: The tests are based on the principal components analyses with 12,898 population structure inference SNPs.

a PLCOca-PLCOco is the original PLCO prostate cancer study.

b PLCOca-NHSco is the reconstructed study with prostate cancer cases from the PLCO, and external controls from NHS.

c NHSca-NHSco is the original NHS breast cancer study.

d NHSca-PLCOco is the reconstructed study with breast cancer cases from the NHS, and external controls from PLCO.

### Population structure in the other three combinations of cases and controls

An analysis of the 12,898 structure inference SNPs in the original breast cancer study (NHSca-NHSco) as well as the two reconstructed studies using external controls (PLCOca-NHSco and NHSca-PLCOco) demonstrated that there were at least 3 PC directions with highly significant large genetic variations (Table 1). The PCA with the second set of 7,017 structure inference SNPs indicates that there are three major PCs (Tracy-Widom test P-value <0.05) in NHSca-NHSco and NHSca-PLCO but only two major PCs in PLCOca-NHSco (Table S3). The estimated PCs along each major direction (the first three for NHSca-NHSco and NHSca-PLCOco, the first two for PLCOca-NHSco) are highly correlated with the counterparts estimated by the set of 12,898 SNPs (Spearman rank correlation coefficient >0.26 and P-value <10^−15^).

### Population structure comparison between PLCO prostate cancer and NHS breast cancer studies

To compare the population substructure between the original two CGEMS initial scans, the PLCOca-PLCOco and NHSca-NHSco, we identified the PC directions by applying the PCA on each study separately, and compared directions between two studies using the Spearman rank correlation coefficient of the SNP loadings (Table 2). The top three PC directions between the two studies are significantly correlated (with Spearman rank correlation coefficient >0.14, and P-value <10^−15^).

**Table 2:** Spearman rank correlation coefficients between pairs of principal component directions from the original PLCO prostate cancer and NHS breast cancer studies.

	NHS PC #1	NHS PC #2	NHS PC #3	NHS PC #4	NHS PC #5
PLCO PC #1	0.73	0.05	0.01	0.00	0.00
PLCO PC #2	0.04	0.51	0.03	0.02	0.00
PLCO PC #3	0.01	0.03	0.14	0.06	0.00
PLCO PC #4	0.02	0.00	0.10	0.05	0.03
PLCO PC #5	0.00	0.00	0.00	0.00	0.00

**Figure 2:**
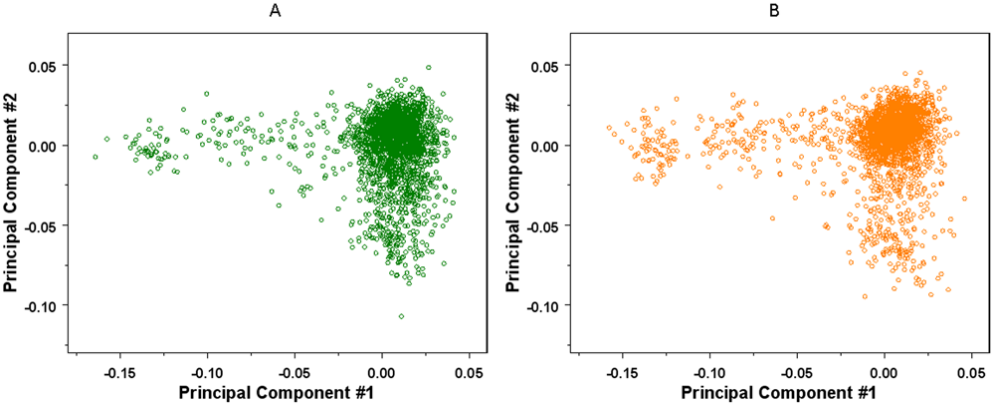
Samples represented by their first two principal components. Principal components (PC, the 1^st^ along the horizontal direction, the 2^nd^ along the vertical direction)) were obtained by applying the PCA on the joint sample of PLCO prostate cancer and NHS breast cancer studies. A) First two PCs for subjects from the PLCO prostate cancer study. B) First two PCs for subjects from the NHS breast cancer study.

We performed a PCA on the joint sample including subjects from both studies. A representation of each subject by its first 2 PCs in a scatter plot stratified by the study (PLCO or NHS) is shown (Figure 2). Visual inspection of Figure 2A and 2B indicates that patterns of population structure of the two studies are indeed similar in the plane of the first 2 PCs. However, further scrutiny reveals very significant difference between the two studies. Between-studies comparisons using the Wilcoxon rank-sum test suggest that the subjects from the two studies have significantly different distributions (with P-values <10^−4^) along each of the top 4 PC directions.

In summary, the comparable patterns in population substructure for the two genome-wide scans in PLCO and NHS suggest that controls from one study may reliably be used to contrast with cases from the other study in an association analysis. This similarity is remarkable since the PLCO prostate cancer study was assembled from male volunteers of European origin participating in a cancer screen trial and enrolled at 10 screening centers while the NHS breast cancer study was based on a sample of female registered nurses participating in a long-term epidemiology study originally enrolled from 14 large US states. However, because we have also shown that the two studies have demonstrable genetic background differences, using external controls in association studies requires a careful examination of the confounding effect due to PS. The observed genetic background difference between the two studies could in part be due to the difference in geographic locations of the source populations which were sampled, as people from distinct regions tend to have different genetic background. For example, by using the Kruskal-Wallis test [29], which is the non-parametric version of the ANOVA test, we find that several major PCs in each study have significantly different distributions across different geographic locations (defined by either the recruitment center in the PLCO prostate cancer study, or the state of residence in the NHS breast cancer study).

### Evidence of confounding by PS

Although we see evidence of at least three axes with large genetic variations in each of the four combinations of cases and controls considered here, confounding by PS would occur only when cases and controls distribute differently along one or more main axes of variations. To assess confounding by PS in each study, we compared the distribution of the cases and controls for each of the top 5 PC directions by using the Wilcoxon rank-sum test (Table 3). The analysis was restricted to the first 5 PC directions in each study since all other directions were not significant by the Tracy-Widom test.

**Table 3:** Principal component comparisons (P-values) between cases and controls based on the Wilcoxon rank-sum test.

	PLCOca-PLCOco^a^	PLCOca-NHSco^b^	NHSca-NHSco^c^	NHSca-PLCOco^d^
PC #1	0.294	4.5×10^−8^	0.664	4.3×10^−6^
PC #2	0.871	2.2×10^−7^	0.289	6.9×10^−12^
PC #3	0.340	0.282	0.036	4.0×10^−3^
PC #4	0.588	1.2×10^−4^	0.015	0.191
PC #5	0.490	0.385	0.943	0.157

Note: For each case-control combination, the Wilcoxon rank-sum tests are based on principal components estimated with 12,898 population structure inference SNPs.

a PLCOca-PLCOco is the original PLCO prostate cancer study.

b PLCOca-NHSco is the reconstructed study with prostate cancer cases from the PLCO, and external controls from NHS.

c NHSca-NHSco is the original NHS breast cancer study.

d NHSca-PLCOco is the reconstructed study with breast cancer cases from the NHS, and external controls from PLCO.

For the two initial scans, PLCOca-PLCOco and NHSca-NHSco, no significant difference (after multiple comparison adjustments) was observed between cases and controls with respect to the distribution on each of the top 5 PC directions (Table 3). Thus, a strong confounding effect by PS in either study is not to be expected, even though PC directions with significantly large genetic variation are evident in these studies. For the two reconstructed studies with external controls, i.e., PLCOca-NHSco and NHSca-PLCOco, cases and external controls display significantly different distributions along the first two PC directions. There is also a third direction (the 4^th^ PC direction in PLCOca-NHSco, and the 3^rd^ PC direction in NHSca-PLCOco) along which cases and external controls differ, although to a lesser extent. Thus, compared with the two studies with internal controls, these findings suggest that the confounding effects by PS are accentuated when controls are culled from an independent study.

**Figure 3:**
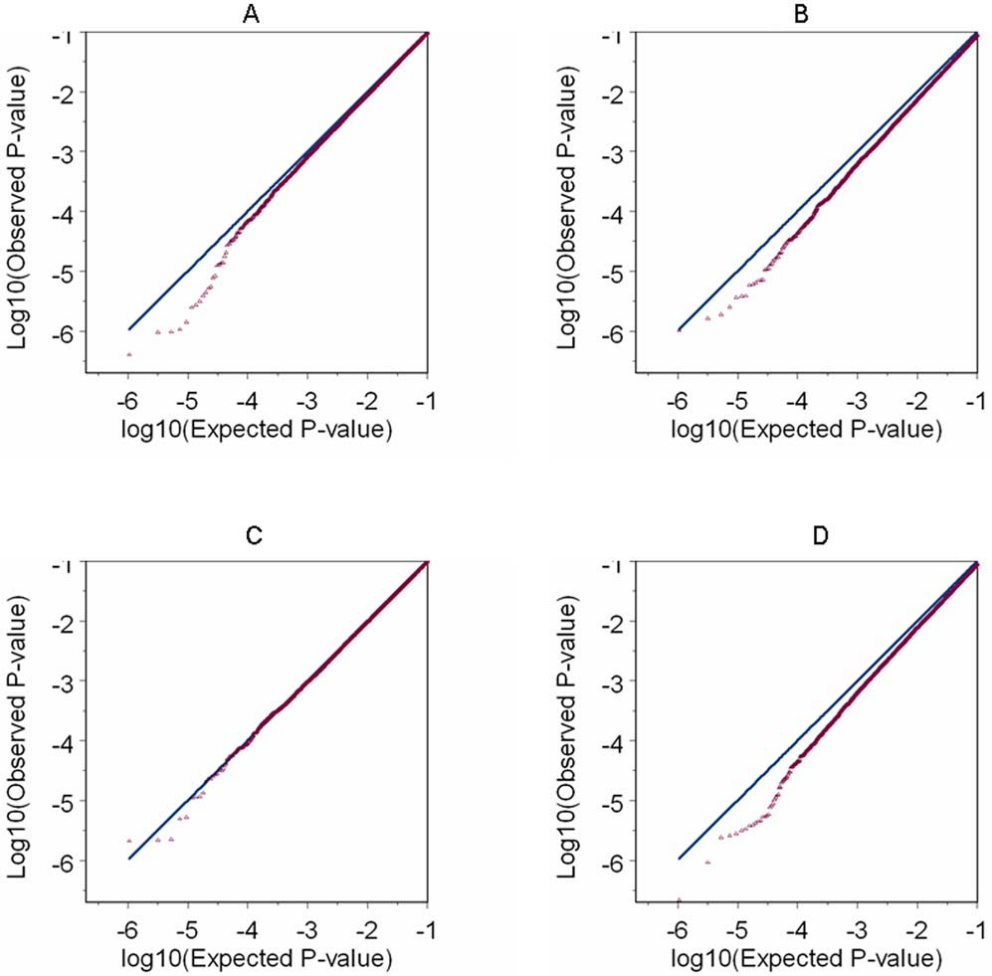
Q-Q plot based on the test without PC adjustment. For each of the four analyses, the Q-Q plot is based on P-values (in log10 scale) that correspond to the 1 d.f. Wald test on 475,116 testing autosomal SNPs by assuming an additive risk model (in logit scale) and without PC adjustment. A) Results for the original prostate cancer study (prostate cancer cases and controls from PLCO). B) Result for the reconstructed prostate cancer study using external controls (prostate cancer cases from PLCO, and external controls from NHS). C) Results for the original breast cancer study (breast cancer cases and controls from NHS). D) Results for the reconstructed breast cancer study using external controls (breast cancer cases from NHS, and external controls from PLCO).

Confounding by PS could lead to an inflated type I error rate for the association test. For each of the four combinations of cases and controls, assuming an additive genetic model we performed a 1-df Wald test (without adjusting for PS) using the standard logistic regression for each testing autosomal SNP. Table 4 shows the over-dispersion factor and empirical type I error (assuming the vast majority of testing SNPs are disease unrelated) under various significance levels. All estimates presented in Table 4 are based on tests performed with the same set of 241,238 genomic control SNPs. They are close to estimates obtained when all 475,116 testing SNPs were used (results not shown). As expected, the two reconstructed studies with external controls have larger over-dispersion factors and higher empirical type I errors compared with the original studies with internal controls. Similar conclusion can be reached based on the Q-Q plot (on the log scale) comparison in Figure 3. From Table 4, the over-dispersion factor is positively correlated with empirical type I errors under various considered significant levels. The over-dispersion factor is indeed a good summary indicating the level of inflation in type I error, as well as the extent of confounding effect. This justifies the use of over-dispersion factor in the permutation procedure.

**Table 4:** Over-dispersion factors and empirical type I errors for the association test without the correction of PS.

		Significance level		
Study	Over-dispersion	0.05^e^	0.01^e^	0.001^e^
NHSca-NHSco^a^	1.005	0.0505	0.0100	0.0010
PLCOca-PLCOco^b^	1.025	0.0527	0.0110	0.0012
NHSca-PLCOco^c^	1.062	0.0572	0.0120	0.0013
PLCOca-NHSco^d^	1.090	0.0588	0.0127	0.0016

Note: The over-dispersion factor and empirical type I errors under various significant levels are estimated by applying the 1-df Wald test statistic without the correction of PS on the set of 241,238 genomic control SNPs. We assume all genomic control SNPs are disease unrelated.

a NHSca-NHSco is the original NHS breast cancer study.

b PLCOca-PLCOco is the original PLCO prostate cancer study.

c NHSca-PLCOco is the reconstructed study with breast cancer cases from the NHS, and external controls from PLCO.

d PLCOca-NHSco is the reconstructed study with prostate cancer cases from the PLCO, and external controls from NHS.

e Empirical type I error rates under the given significant level (assuming all genomic control SNPs are disease-unrelated).

### PC adjustment and its impact on over-dispersion factor

Following Price et al. [15], the confounding effect of PS can be corrected by adjusting for a defined set of PCs. The typical method adjusts for *L* top-ranked PCs, with *L* being either pre-determined (e.g., 10), or the number of significant PCs (e.g., those with P-value <0.05 based on the Tracy-Widom test). To evaluate this strategy, we estimated over-dispersion factors for association tests (1 d.f. Wald test) based on results on the set of 241,238 genomic control SNPs with adjustment for varying numbers (from 1 to 10) of top ranked PCs (Table 5). We observe that the over-dispersion factor does not necessarily decrease as the number of adjusted PCs increases. Table 5 also suggests that adjusting non-significant PCs does not appear to have a major impact on the inflation reduction

**Table 5:** Over-dispersion factor (and the empirical type I error under the significant level of 0.05) for association tests with adjustment for various numbers of PCs.

PCs chosen for the adjustment	PLOCca-PLCOco^a^	PLCOca-NHSco^b^	NHSca-NHSco^c^	NHSca-PLCOco^d^
0 PC	1.025 (0.053)	1.090 (0.059)	1.005 (0.051)	1.062 (0.057)
1^st^ PC	1.020 (0.052)	1.055 (0.055)	1.006 (0.050)	1.040 (0.055)
1–2 PCs	1.022 (0.052)	1.040 (0.053)	1.004 (0.050)	1.013 (0.052)
1–3 PCs	1.021 (0.052)	1.040 (0.053)	1.005 (0.050)	1.006 (0.052)
1–4 PCs	1.021 (0.052)	1.032 (0.053)	1.005 (0.050)	1.007 (0.052)
1–5 PCs	1.023 (0.052)	1.032 (0.053)	1.006 (0.050)	1.008 (0.052)
1–6 PCs	1.024 (0.052)	1.032 (0.053)	1.007 (0.050)	1.010 (0.052)
1–7 PCs	1.025 (0.052)	1.033 (0.053)	1.008 (0.050)	1.010 (0.051)
1–8 PCs	1.025 (0.052)	1.036 (0.053)	1.007 (0.051)	1.011 (0.052)
1–9 PCs	1.025 (0.053)	1.036 (0.053)	1.007 (0.051)	1.011 (0.052)
1–10 PCs	1.025 (0.053)	1.036 (0.053)	1.008 (0.051)	1.010 (0.052)
Selected PCs^e^	1.020 (0.052)	1.032 (0.053)	1.003 (0.050)	1.006 (0.052)

Note: The over-dispersion factor (and the empirical type I error under the significant level of 0.05) are estimated by applying the 1-df Wald test statistic with the adjustment for selected PCs on the set of 241,238 genomic control SNPs.

a PLCOca-PLCOco is the original PLCO prostate cancer study.

b PLCOca-NHSco is the reconstructed study with prostate cancer cases from the PLCO, and external controls from NHS.

c NHSca-NHSco is the original NHS breast cancer study.

d NHSca-PLCOco is the reconstructed study with breast cancer cases from the NHS, and external controls from PLCO.

e Those PCs were chosen by the proposed permutation procedure for the correction of PS. The selected sets of PCs for individual analyses (from left to right) are: 1^st^ PC; 1^st^, 2^nd^, and 4^th^ PCs; 2^nd^ PC; and 1^st^, 2^nd^, and 3^rd^ PCs. The PCs were ordered according to their corresponding eigenvalues

The following simulation further supports this observation. For each of the four considered studies, we rearranged each subject's case/control status so that new designated case and control groups were completely separately on a chosen PC direction (e.g., the first PC direction), and evaluated the over-dispersion factor and empirical type I error rate in the new generated dataset. Results are summarized in Table S4). When the mismatch (to the most extreme extend) between cases and controls occurred on a PC with large genetic variation, the resultant inflation in type I error was much more severe than the one observed when the disparity occurred on a PC with relatively low variation (high Tracy-Widom test P-value). For all 4 considered studies, the over-dispersion factor was around or less than 1.05 when the mismatch (in the worst scenario) of cases and controls occurred on a PC with the associated Tracy-Widom test P-value larger than 0.05.

Using 0.05 as the P-value threshold for selecting PCs with large variation, four or five PCs were chosen for the four studies under investigation (Table 1). Moreover, results from Table 5 suggest that adjustment of a PC with significantly large variation may not necessarily reduce the over-dispersion factor if cases and controls have comparable distribution over that PC. For example, in the original prostate cancer scan, the cases and controls have very similar genetic background as seen in the 2^nd^ PC direction (see Table 5). In this case, compared to adjustment of the first PC, adding the 2^nd^ PC into the adjustment does not reduce the over-dispersion factor.

To minimize the number of adjusted PCs while allowing an effective correction of the confounding effect by PS, our proposed selection procedure identifies PCs that are effective in the reduction of the over-dispersion factor. A PC is chosen for the correction of PS whenever the over-dispersion factor in the model with the PC included is significantly lower than the expected value of the factor from including an additional randomly generated covariate. When applied to the two original studies, this new procedure identified a single PC to achieve the optimal PS correction. Interestingly, the procedure selected the first PC (corresponding to the largest genetic variation) for the PLCO prostate cancer study, but the second PC for the NHS breast cancer study suggesting that the control selection procedure used in the NHS study effectively removed the confounding that might have been caused by the axis with the largest genetic variation. When the controls were exchanged between the two studies, the procedure required the 1^st^, 2^nd^, and 4^th^ PCs in PLCOca-NHSco, and the 1^st^, 2^nd^, and 3^rd^ PCs in NHSca-PLCOco. Thus, the increased heterogeneity between cases and controls created in the two reconstructed studies resulted in the inclusion of two additional PCs in order to sufficiently correct for the confounding effect.

Compared to the strategy of adjusting for PCs with large genetic variation, the proposed permutation procedure picks fewer PCs and reduces the over-dispersion factor to a similar or even lower level (Table 5). With either strategy, the over-dispersion factor is slightly decreased from its uncorrected level in the two original studies with internal controls. In the two reconstructed analyses, the over-dispersion level can be reduced substantially and an improved fitting of the Q-Q plot to the diagonal can be achieved. Comparison of Figures 3 with 4 demonstrates the conspicuous effect of PC adjustment in the two reconstructed analyses as this effect is hardly visible on the two original studies with internal controls.

**Figure 4:**
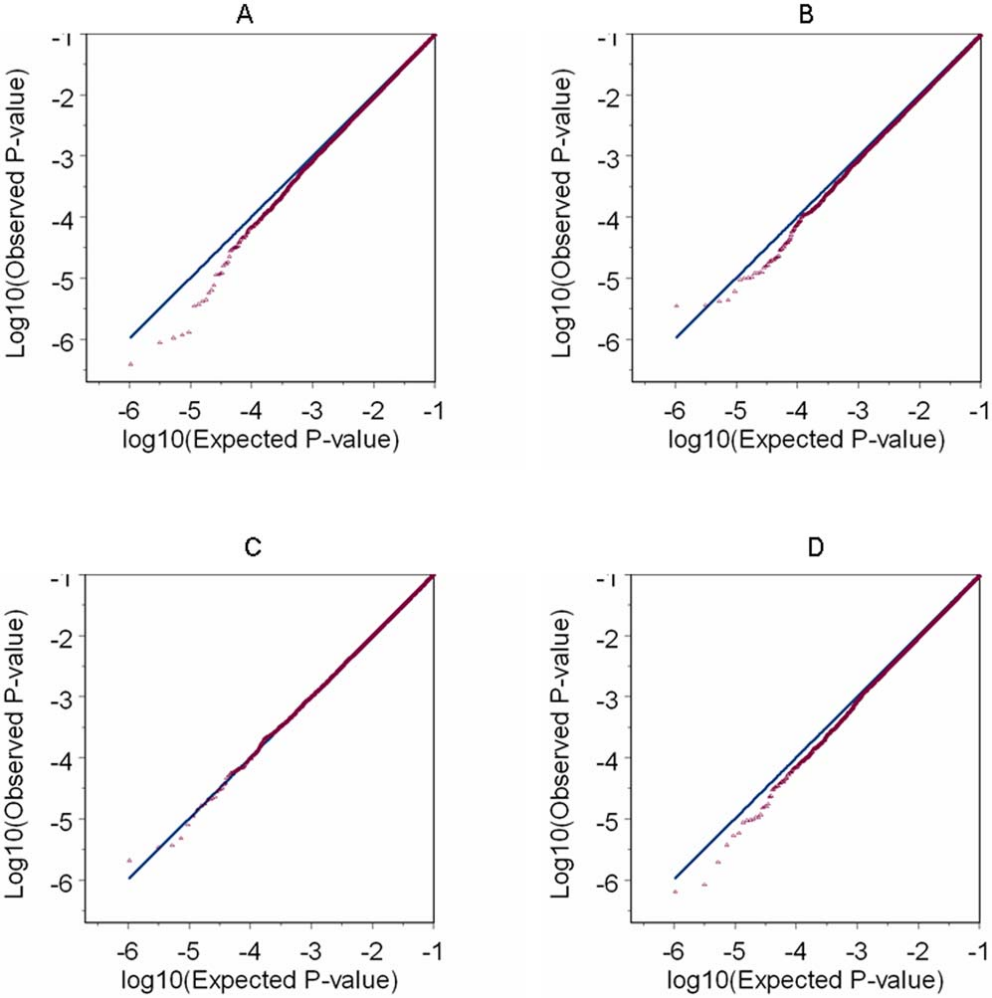
Q-Q plot based on the test with PC adjustment. For each of the four analyses, the Q-Q plot is based on P-values (in log10 scale) that correspond to the 1 d.f. Wald test on 475,116 testing autosomal SNPs by assuming an additive risk model (in logit scale) and with PC adjustment. The PCs used in adjustment are selected by the proposed permutation procedure. A) Results for the original prostate cancer study (prostate cancer cases and controls from PLCO). B) Results for the reconstructed prostate cancer study using external controls (prostate cancer cases from PLCO, and external controls from NHS). C) Results for the original breast cancer study (breast cancer cases and controls from NHS). D) Results for the reconstructed breast cancer study using external controls (breast cancer cases from NHS, and external controls from PLCO).

### The impact of PC adjustment on SNP ranking and selection for follow-up study

**Figure 5:**
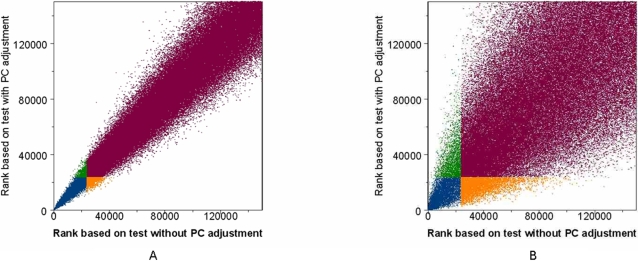
SNP ranking correlation in prostate cancer studies. In each plot, SNPs' rankings based on the 1 d.f. Wald test on 475,116 testing autosomal SNPs without PC adjustment are compared with their rankings based on the 1 d.f. Wald test with adjustment for PCs chosen by the permutation procedure. The SNPs in blue are ranked among the top 5% by tests both with and without PC adjustment. The SNPs in green and orange are ranked among the top 5% by only one of the tests. A) Results based on the original prostate cancer study (prostate cancer cases and controls from PLCO). The 1^st^ PC was chosen for PS correction. B) Results based on the reconstructed prostate cancer study using external controls (prostate cancer cases from PLCO, and external controls from NHS). The 1^st^, 2^nd^ and 4^th^ PCs were chosen for PS correction.

**Figure 6:**
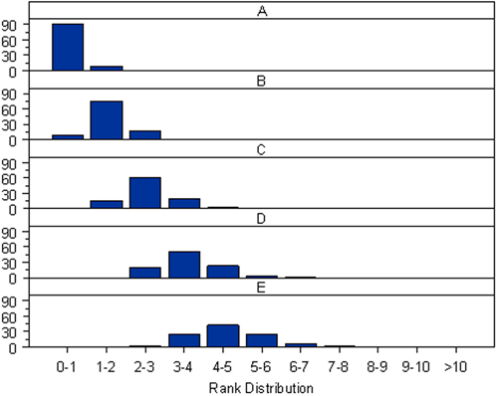
The conditional ranking distribution for the original PLCO prostate cancer study. Each plot shows the histogram of ranks according to the test without PC adjustment for SNPs ranked within a given range by the test with the adjustment for the 1^st^ PC (chosen by the proposed permutation procedure). The ranking ranges (%) are shown on the horizontal axis. The frequencies (%) are shown on the vertical axis. A) The histogram of ranks for SNPs ranked in the top 0–1% by the test with PC adjustment. B) The histogram of ranks for SNPs ranked in the top 1–2% by the test with PC adjustment. C) The histogram of ranks for SNPs ranked in the top 2–3% by the test with PC adjustment. D) The histogram of ranks for SNPs ranked in the top 3–4% by the test with PC adjustment. E) The histogram of ranks for SNPs ranked in the top 4–5% by the test with PC adjustment.

**Figure 7:**
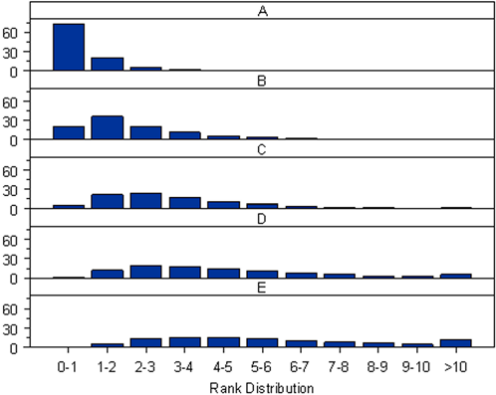
The conditional ranking distribution for the reconstructed prostate cancer study using external controls. Each plot shows the histogram of ranks according to the test without PC adjustment for SNPs ranked within a given range by the test with the adjustment for the 1^st^, 2^nd^, and 4^th^ PCs (chosen by the proposed permutation procedure). The ranking ranges (%) are shown on the horizontal axis. The frequencies (%) are shown on the vertical axis. A) The histogram of ranks for SNPs ranked in the top 0–1% by the test with PC adjustment. B) The histogram of ranks for SNPs ranked in the top 1–2% by the test with PC adjustment. C) The histogram of ranks for SNPs ranked in the top 2–3% by the test with PC adjustment. D) The histogram of ranks for SNPs ranked in the top 3–4% by the test with PC adjustment. E) The histogram of ranks for SNPs ranked in the top 4–5% by the test with PC adjustment.

In a multiple-stage association study [30], the number of SNPs to be taken into the follow-up steps is usually fixed in advance by cost considerations so that the SNPs are selected based on the ranking of their test statistics rather than their P-values. In CGEMS, the number of SNPs to be taken in follow-up 1 was approximately 5% of those used in the initial genome wide scan [21]. Consequently, adjusting for PCs in the association test would be expected to change the ranking of the SNPs. In Figures 5, 6 and 7 we show the rank shuffling among the set of 475,116 testing SNPs in the prostate cancer study with internal controls (PLCOca-PLCOco) and with external controls (PLCOca-NHSco). In either case, PCs were selected by the proposed permutation procedure. A similar pattern can be observed in the breast cancer studies with internal and external controls (results shown in Figures S3, S4, S5). Comparing Figure 5A with 5B shows the high correlation of the two sets of ranks in the original study with internal controls and the lower correlation in the reconstructed studies using external controls. Figures 6 and 7 show the histogram of ranks according to the test without PC adjustment for SNPs ranked within a given range by the test with PC adjustment. Again, the discordance between two ranks in the analyses with external controls is more prominent than in the original study.

On the premise that the top 5% of the testing SNPs ranked by the statistic using a given PC adjustment strategy are to be followed-up to the next stage, we evaluated the concordance between two sets of follow-up SNPs, namely, those chosen by the test adjusting for PCs selected by the permutation procedure, and those chosen by the test adjusting for PCs selected by an alternative strategy (such as adjusting for the top 10 PCs, or no PC adjustment). In PLCOca-PLCOco, among the follow-up SNPs chosen by test without PC adjustment, approximately 7% of them would not have been chosen by the test with the adjustment of PCs identified by the permutation procedure. The discrepancy increases to 23% in the corresponding study with external controls PLCOca-NHSco (Table 6). A similar pattern was also observed in the breast cancer studies with swapping of internal and external controls. We note that in each of the four studies considered the vast majority of SNPs ranked in the top 1% by the test with PC adjustment were ranked within the top 5% by the test without PC adjustment (Figures 6A, 7A, S5A, and S6A). Figures 5 and S3 show that both ranks of the most discordant SNPs are close to the threshold.

**Table 6:** Discrepancy in SNP selection for the follow-up study between the permutation procedure and an alternative PC adjustment strategy.

PC adjustment Strategy	PLOCca-PLCOco^a^	PLCOca-NHSco^b^	NHSca-NHSco^c^	NHSca-PLCOco^d^
0 PC adjustment^e^	7.3%	22.8%	5.6%	22.6%
Adjustment for top 10 PCs^f^	4.5%	6.3%	9.4%	6.4%
Adjustment for Significant PCs^g^	3.7%	2.6%	8.2%	4.0%

Note: Under the premise that the top 5% of the testing SNPs ranked by the statistic using a given PC adjustment strategy are to be followed-up to the next stage, the discrepancy the proportion of SNPs selected by this procedure and not selected by the permutation procedure.

a PLCOca-PLCOco is the original PLCO prostate cancer study.

b PLCOca-NHSco is the reconstructed study with prostate cancer cases from the PLCO, and external controls from NHS.

c NHSca-NHSco is the original NHS breast cancer study.

d NHSca-PLCOco is the reconstructed study with breast cancer cases from the NHS, and external controls from PLCO.

e No PC adjustment in the association test.

f Adjustment for the top 10 PCs ranked by their associated eigenvalues.

g Adjustment for the PCs with significant genetic variation (P-value <0.05 by the Tracy Widom test).

Compared to the proposed permutation procedure, strategies that would have taken the top 10 PCs or only the significant PCs (P-value <0.05 based on the Tracy-Widom test) would have changed the selection of the follow-up SNPs by an average of 6.7% or by an average of 4.6% among the four considered studies (Table 6).

### Numbers of SNPs needed for the PCA in GWAS

To empirically evaluate the relationship between the reduction in the over-dispersion factor and the number of structure inference SNPs used in the correction of PS, we focused on the reconstructed study PLCOca-NHSco, where the most extensive confounding by PS was observed (*λ* = 1.090). We conducted PCA using nested sets of 1,500, 3,000, 6,000, 9,000 and 12,898 population structure inference SNPs (Table 7).

**Table 7:** Detection and correction for population stratification using various numbers of SNPs for PCA in the reconstructed study comparing prostate cancer cases from PLCO with controls from NHS.

Number of SNPs used in PCA^a^	PCs with large genetic variation^b^	PCs associated with outcome^c^	PCs chosen for PS correction^d^	Over-dispersion factor after PS correction^e^
1,500	1^st^, 2^nd^	none	1^st^	1.067
3,000	1^st^, 2^nd^	1^st^, 2^nd^	1^st^, 2^nd^	1.052
6,000	1^st^, 2^nd^	1^st^, 2^nd^	1^st^, 2^nd^	1.043
9,000	1^st^, 2^nd^, 3^rd^	1^st^, 2^nd^	1^st^, 2^nd^	1.039
12,898	1^st^, 2^nd^, 3^rd^, 4^th^	1^st^, 2^nd^, 4^th^	1^st^, 2^nd^, 4^th^	1.032

a SNPs are randomly (except that the large set contains the smaller set) selected from the panel of 12,898 PS inference SNPs that are common to both the Illumina and Affymetrix commercial platforms.

b These PCs have a significantly large genetic variation (with P-values less than 0.05 based on the Tracy Wildom test).

c These PCs have a significantly different distribution between case and control groups (with P-value less than 0.05 based on the Wilcoxon rank-sum test).

d These PCs were chosen by the proposed permutation procedure for the correction of PS.

e The over dispersion factor was estimated by applying the 1-df Wald test statistic with the adjustment for the chosen PCs on the set of 241,238 genomic control SNPs.

Table 7 shows that, as expected, using a larger number of SNPs in the correction of PS results in the detection of more subtle population structure. For example, with 12,898 SNPs, we can detect four significant PCs (P-value <0.05 based on the Tracy-Widom test). With 6,000 or fewer population structure inference SNPs, only two significant PCs can be identified. Also, the over-dispersion factor of the test with the adjustment of PCs chosen by the proposed permutation procedure decreases as the number of SNPs used in the PCA increases. When 12,898 SNPs are used in the correction of PS, we can reduce the over-dispersion factor to an acceptable level (*λ* = 1.032) with the adjustment of 3 PCs.

### The importance of choosing SNPs with low background LD for PCA

The set of structural inference SNPs we chose for the PCA consists of 12,898 SNPs that had low background LD measured in the joint PLCO and NHS control samples (*r* ^2^ less than 0.004 for any pair located within 500 kb on the same chromosome). The restriction to SNPs with very low local pairwise correlation ensures that the PCA findings reflect the genome-wide variation pattern, and are not overly influenced by regional LD pattern. WTCCC also adopted this strategy [20].

To demonstrate the importance of the selection of SNPs with low background LD, we conducted PCA on the study PLCOca-NHSco using all 12,536 autosomal SNPs located on chromosome 20 without filtering out SNPs in high LD, and summarized the results in Table S5 and Figure S6. By inspecting the SNP loadings on a given PC direction, it is evident that each of the top 5 PC directions is heavily driven by regional LD patterns (Figure S6). Thus, it is not surprising to see from Table S5 that the over-dispersion is still high even after the adjustment of the top 10 PCs, in contrast to the results shown in Table 5.

## Discussion

We used empirical data from two GWAS within the CGEMS project to assess the extent and impact of PS in studies with two distinct control selection strategies. We also evaluated our proposed procedures for choosing structural inference SNPs as well as for selecting the PCs for correction of PS. In the two original GWAS based on the nested case-control design, we observed only minor confounding effect by PS with over-dispersion factors of 1.025 and 1.005 for prostate and breast cancer studies, respectively. These small inflations, which in practice may not raise major concern, can be further reduced by adjustment for a single PC. In the two reconstructed studies where cases and controls were collected independently using different designs, we observed more extensive confounding effect by PS with over-dispersion factors of 1.090 and 1.062. In these studies with external controls, three principal components were required to optimally correct for the confounding effect of PS, resulting in the reduction of the inflation factor to a level comparable to that in the original nested case-control studies. Our conclusions are based on two actual studies of two cancer sites and two hypothetical ones using observed data in the European American populations. The impact of PS in other populations, such as African Americans, may be different and thus, requires independent assessment.

Case-control studies nested in prospective cohorts, such as the two original GWAS in the CGEMS project, tend to minimize biases introduced when cases and controls are selected from different populations. We found that cases and controls had comparable genetic background and only minor confounding effect by PS in these two studies. In stand-alone case-control studies, which are not nested within a cohort, the bias is likely to be somewhat greater because of difficulties in control selection when there is no roster of the underlying population producing the cases.

A more extreme but convenient and cost-efficient design alternative, notably taken recently by the WTCCC [20], is the use of external controls that are collected independently with little reference to the population from which cases are selected. The large number of disease-unrelated SNPs measured in a GWAS can be utilized to evaluate and when applicable, correct for the confounding effect induced by the genetic ancestral disparity between the case and control groups. Therefore, the stringent requirement of control selection imposed according to the classical epidemiology paradigm could be relaxed to some extent. This view is supported by our analyses of two reconstructed studies with independently collected controls. It appears that an appropriate PC adjustment can effectively correct for the elevated confounding effect introduced by the use of less desirable controls.

Adjusting for unnecessary covariates incurs the risk of decreasing power [31]. We have presented a simulation to demonstrate that the unnecessary adjustment of population substructure (even one PC) could lead to a significant loss in power (Text S1, Table S1). A permutation procedure is proposed to identify the minimal number of PCs while allowing an effective correction of the confounding effect. By applying this new procedure to the two original GWAS with internal controls and two reconstructed studies with external controls, we documented its advantage over other commonly used PC selection strategies. At the expense of computing time, the new procedure is able to pick fewer PCs while reducing the over-dispersion factor to a similar or even lower level.

The identified set of 12,898 SNPs with low background LD in European American population and common to both the Illumina and Affymetrix commercial platforms can be used in PCA for evaluation of population structure. We detected similar patterns of population substructure in the original scans even though they were nested within different cohorts. The top three axes from the two independent studies appear to point to similar directions and are likely to be a characteristic of the European American population. Further studies are required to correlate differences along the axes of genetic variation with groups defined by self-described ethnic background, geographic location or specific demographic histories. Based on our present experiences, we believe that this set of SNPs should be sufficient for the inference and correction of population structure in GWAS conducted using either the Illumina or Affymetrix commercial platforms within the European American populations, and enables the comparison of population structure between studies performed on different platforms without relying on genotype imputation. The same search algorithm can be used to identify structure inference SNPs suitable for GWAS in other populations, such as African Americans.

In the replication stages of a multi-stage GWAS, it would be impractical to genotype the entire list of 12,898 SNPs for the correction of PS. In the process of selecting a fixed number of SNPs for the follow-up study that would typically involve 10,000 to 50,000 SNPs, there is always a trade-off between the number of SNPs allocated for population structure inference and the number of candidate disease-associated SNPs chosen for the validation/replication. Recently, Price et al. [32] and Tian et al. [33] identified panels of SNPs that are informative for discerning major European ancestries in European American populations. For example, Price et al. [32] designed a panel of 300 SNPs that aims to distinguish northwest European, southeast European, and Ashkenazi Jewish ancestry. These panels of ancestral informative SNPs are potentially useful in replication studies with a similar anticipated population substructure, but may not be as robust in studies where the population sub-structure may be different or unknown. Rapid accumulation of GWAS and their replication studies should provide ample opportunities for designing and validating panels of ancestral informative markers targeting various stratified or admixed populations.

Our analysis has focused on the confounding effect of PS on single-marker association analyses. While there is an increasing emphasis on detecting interactions between genes and between genes and the environment, Wang et al. [34] recently evaluated the bias resulting from the confounding effect of PS in studies of gene-gene or gene-environment interactions. Based on simulation studies, they showed that bias due to PS could be large for studies of interactions, especially when strong correlation between genes (or between genetic and environmental factors) takes place. Using data generated from the CGEMS project and tools developed in this paper, we can empirically evaluate the impact of PS on the study of gene-gene interaction under different control selection strategies. However, valid assessment of effect of PS on gene-environment interaction may require additional assumptions depending on the control selection procedure chosen.

There are several additional issues other than the type I error inflation arising from PS to consider when evaluating the appropriateness of convenience controls versus controls selected to reflect the study-base that produced the cases. There may be differential genotyping error between cases and controls due to variation in the processing of biological samples. Also, selection bias for non-genetic covariates that can not be corrected by PCA could lead to misleading estimates of interactions [35]. The selection of cases and controls from a common prospective cohort tends to minimize potential discrepancies.

The analyses of empirical data generated from the CGEMS project suggest that the effect of PS in the GWAS of prostate and breast cancers conducted in European American is small when the study is epidemiologically well designed, but can be substantial when controls and cases are drawn from separate studies. The elevated confounding effect of PS due to the use of less desirable controls can be effectively mitigated by methods such as the one proposed here. The impact of using convenience controls on the power for the detection of disease related markers needs to be further investigated, especially in recently admixed populations.

## Supporting Information

Text S1 An example demonstrating the loss in power due to unnecessary adjustment of population substructure
(0.09 MB DOC)

Table S1 Power comparison between the association tests with and without population substructure adjustment
(0.03 MB DOC)

Table S2 List of 12,898 population inference SNPs
(1.13 MB XLS)

Table S3 Tracy-Widom tests and associated P-values (in parenthesis) for the significance of principal components based on 7,017 structure inference SNPs
(0.03 MB DOC)

Table S4 Over-dispersion factor (and the empirical type I error under the significant level of 0.05) when cases and controls are reassigned so that they are completely separated along a chosen PC direction.
(0.03 MB DOC)

Table S5 Results on the PLCOca-NHSco using the set of 12,536 SNPs on chromosome 20 in the principal components analysis
(0.03 MB DOC)

Figure S1 Distribution of the minor allele frequency of 12,898 population substructure inference SNPs on each autosome. Each SNP's physical map position on the chromosome is based on the reference genome build 36.2 and the latest dbSNP build 128. One SNP (rs3789771) has no map information and is excluded from the figure.
(0.22 MB TIF)

Figure S2 SNP Loadings from PCA using 12,898 population substructure inference SNPs in the PLCO prostate cancer study. SNPs are organized according to the order of their chromosome positions.
(0.12 MB TIF)

Figure S3 SNP ranking correlation in breast cancer studies. In each plot, SNPs' rankings based on the 1 d.f. Wald test on 475,116 testing autosomal SNPs without PC adjustment are compared with their rankings based on the 1 d.f. Wald test with adjustment for PCs chosen by the permutation procedure. The SNPs in blue are ranked among the top 5% by tests both with and without PC adjustment. The SNPs in green and orange are ranked among the top 5% by only one of the tests. A) Results based on the original breast cancer study (breast cancer cases and controls from NHS). The 2nd PC was chosen for PS correction. B) Results based on the reconstructed breast cancer study using external controls (breast cancer cases from NHS, and external controls from PLCO). The 1st, 2nd and 3rd PCs were chosen for PS correction.
(0.29 MB TIF)

Figure S4 The conditional ranking distribution for the original NHS breast cancer study. Each plot shows the histogram of ranks according to the test without PC adjustment for SNPs ranked within a given range by the test with the adjustment for the 2nd PC (chosen by the proposed permutation procedure). The ranking ranges (%) are shown on the horizontal axis. The frequencies (%) are shown on the vertical axis. A) The histogram of ranks for SNPs ranked in the top 0–1% by the test with PC adjustment. B) The histogram of ranks for SNPs ranked in the top 1–2% by the test with PC adjustment. C) The histogram of ranks for SNPs ranked in the top 2–3% by the test with PC adjustment. D) The histogram of ranks for SNPs ranked in the top 3–4% by the test with PC adjustment. E) The histogram of ranks for SNPs ranked in the top 4–5% by the test with PC adjustment.
(0.02 MB TIF)

Figure S5 The conditional ranking distribution for the reconstructed breast cancer study using external controls. Each plot shows the histogram of ranks according to the test without PC adjustment for SNPs ranked within a given range by the test with the adjustment for the 1st, 2nd, and 3rd PCs (chosen by the proposed permutation procedure). The ranking ranges (%) are shown on the horizontal axis. The frequencies (%) are shown on the vertical axis. A) The histogram of ranks for SNPs ranked in the top 0–1% by the test with PC adjustment. B) The histogram of ranks for SNPs ranked in the top 1–2% by the test with PC adjustment. C) The histogram of ranks for SNPs ranked in the top 2–3% by the test with PC adjustment. D) The histogram of ranks for SNPs ranked in the top 3–4% by the test with PC adjustment. E) The histogram of ranks for SNPs ranked in the top 4–5% by the test with PC adjustment.
(0.02 MB TIF)

Figure S6 SNP loadings from PCA using SNPs on chromosome 20 in prostate cancer study using external controls. SNPs are organized according to their positions (in base pair) on chromosome 20.
(0.09 MB TIF)
