# Supplementary material for: Population Substructure and Control Selection in Genome-Wide Association Studies
Source: PLoS One. 2008 Jul 2;3(7):e2551. doi: 10.1371/journal.pone.0002551 (PMC2432498; doi:10.1371/journal.pone.0002551)
Supplement: Text S1 — An example demonstrating the loss in power due to unnecessary adjustment of population substructure (0.09 MB DOC) [file pone.0002551.s001.doc]

**An example demonstrating the loss in power due to unnecessary adjustment of population substructure**

We consider an association study of certain disease, say lung cancer, in a population consisting of two equal sized genetically different sub-populations, subpopulation I and subpopulation II. We assume the following disease risk model for both subpopulations,

$\log\frac{p}{1-p}=\alpha_{0}+\beta_{0}X_{G}+\gamma_{0}X_{E}$,

where $X_{E}$ is a recognized environment risk factor, such as smoking, with 1 and 0 representing exposed and unexposed subjects, respectively;$X_{G}$ is the coding variable for the genotype at the true disease risk locus, with 1 for having at least one copy of the disease risk allele, 0 for having none of the disease risk allele. In the above risk model, we assume the same odds ratio $\exp(\beta_{0})=\exp(\gamma_{0})=1.3$ for $X_{E}$ and $X_{G}$. Within each subpopulation, $X_{E}$ and $X_{G}$ are independent. For a subject in subpopulation I, we denote the probability of having $X_{E}=1$ as $f_{1}$, and the probability of having $X_{G}=1$ as $f_{2}$. In sub-population II, those two probabilities are reversed, i.e., the probability of having $X_{E}=1$ is $f_{2}$, and the probability of having $X_{G}=1$ is $f_{1}$. Under the above assumptions, the two subpopulations have the same disease prevalence and thus, there is no population stratification even though the distribution of $X_{G}$ (and $X_{E}$) are different between the two subpopulations. Given $(f_{1},\text{ }f_{2})$, $\beta_{0}$ and $\gamma_{0}$ , we choose the value for $\alpha_{0}$ in the risk model so that the disease prevalence in either subpopulation is always 5%.

Suppose a GWAS is conducted with a sample of 1,000 cases and 1,000 controls that are randomly collected from the study population without recognizing the existence of subpopulations. Because there is no disease prevalence difference between the two subpopulations, there is no confounding due to the existing of population substructure in the study. Through simulations, we compare the power of the following two association tests for detecting the association between the disease risk and the disease locus, one based on the Wald test for $\beta$, the coefficient of $X_{G}$, derived from the following logistic regression model adjusting for the environment risk factor $X_{E}$,

$\log\frac{p}{1-p}=\mu+\beta X_{G}+\gamma X_{E}$,

the other based on the Wald test for $\beta$ derived from the following logistic regression model adjusting for both $X_{E}$ and the estimated subpopulation structure $Z$ (could be the first PC from the PCA).,

$\log\frac{p}{1-p}=\mu+\beta X_{G}+\gamma X_{E}+\eta Z$.

For simplicity, we assume that the two subpopulations are so genetically different that $Z$ is a perfect prediction of the true subpopulation membership.

Power comparison results based on 1,000 replications under each considered configuration of $(f_{1},\text{ }f_{2})$ are given in Table S1. From the table we can see the negative impact of unnecessary adjustment for the population substructure, especially when the difference between $f_{1}$ and $f_{2}$ is large. The drop in power reflects the correlation between $X_{G}$ and $Z$, which leads to an increase of variance in the coefficient estimation for $X_{G}$ upon adjusting for $Z$. This phenomenon can be analytically proven in the framework of general linear model [31]. As the difference between $f_{1}$ and $f_{2}$ gets smaller (i.e., the distribution of $X_{G}$ in the two subpopulations becomes similar), the correlation between $X_{G}$ and $Z$ becomes weaker, so as its negative impact on power.

Our proposed PC selection procedure described in the text is unlikely to choose any PCs among those with Tracy-Widom test P-value less than 0.05 since there is no PS in this consider scenario. Some numerical experiments confirmed this.
