# Supplementary material for: Population Substructure and Control Selection in Genome-Wide Association Studies
Source: PLoS One. 2008 Jul 2;3(7):e2551. doi: 10.1371/journal.pone.0002551 (PMC2432498; doi:10.1371/journal.pone.0002551)
Supplement: Table S1 — Power comparison between the association tests with and without population substructure adjustment (0.03 MB DOC) [file pone.0002551.s002.doc]

**Table S1. Power comparison between the association tests with and without population substructure adjustment**

| (*f*_1_, *f*_2_) ^a^ | Unadjusted^b^ | Adjusted^c^ |
| --- | --- | --- |
| (0.1, 0.9) | 0.63 | 0.43 |
| (0.1, 0.7) | 0.78 | 0.64 |
| (0.1, 0.5) | 0.75 | 0.68 |
| (0.1, 0.3) | 0.66 | 0.64 |
| (0.1, 0.1) | 0.45 | 0.45 |

Note: The simulation experiment is described in Text S1. The significant level is 0.05.

^a^Values for *f*_1_, the probability of being exposed to the environment risk, and for *f*_2_, the probability of having at least one copy of the disease risk allele, for a subject from subpopulation I. These values are reversed for subpopulation II.

^b^The association test based on the Wald statistic derived from the logistic regression model adjusting for the environment risk factor.

^c^The association test based on the Wald statistic derived from the logistic regression model adjusting for both environment risk factor and population substructure.
