# Supplementary material for: Population Substructure and Control Selection in Genome-Wide Association Studies
Source: PLoS One. 2008 Jul 2;3(7):e2551. doi: 10.1371/journal.pone.0002551 (PMC2432498; doi:10.1371/journal.pone.0002551)
Supplement: Table S3 — Tracy-Widom tests and associated P-values (in parenthesis) for the significance of principal components based on 7,017 structure inference SNPs (0.03 MB DOC) [file pone.0002551.s004.doc]

**Table S3. Tracy-Widom tests and associated P-values (in parenthesis) for the significance of principal components based on 7,017 structure inference SNPs**

|  | PLCOca-PLCOco^a^ | PLCOca-NHSco^b^ | NHSca-NHSco^c^ | NHSca-PLCOco^d^ |
| --- | --- | --- | --- | --- |
| PC #1 | 277.99 (<10^-7^) | 311.50 (<10^-7^) | 444.95 (<10^-7^) | 413.99 (<10^-7^) |
| PC #2 | 86.68 (<10^-7^) | 100.58 (<10^-7^) | 72.42 (<10^-7^) | 60.24 (<10^-7^) |
| PC #3 | 0.17 (0.139) | -0.36 (0.243) | 11.10 (<10^-7^) | 14.92 (<10^-7^) |
| PC #4 | -1.49 (0.571) | -1.48 (0.567) | -1.98 (0.720) | -0.34 (0.238) |
| PC #5 | -4.78 (0.999) | -3.27 (0.958) | -3.02 (0.933) | -3.30 (0.961) |

^a^PLCOca-PLCOco is the original PLCO prostate cancer study.

^b^PLCOca-NHSco is the reconstructed study with prostate cancer cases from the PLCO, and external controls from NHS.

^c^NHSca-NHSco is the original NHS breast cancer study.

^d^NHSca-PLCOco is the reconstructed study with breast cancer cases from the NHS, and external controls from PLCO.
