# Supplementary material for: Population Substructure and Control Selection in Genome-Wide Association Studies
Source: PLoS One. 2008 Jul 2;3(7):e2551. doi: 10.1371/journal.pone.0002551 (PMC2432498; doi:10.1371/journal.pone.0002551)
Supplement: Table S4 — Over-dispersion factor (and the empirical type I error under the significant level of 0.05) when cases and controls are reassigned so that they are completely separated along a chosen PC direction. (0.03 MB DOC) [file pone.0002551.s005.doc]

**Table S4. Over-dispersion factor (and the empirical type I error under the significant level of 0.05) when cases and controls are reassigned so that they are completely separated along a chosen PC direction**

| PC chosen for case/control assignment | PLOCca-  PLCOco^a^ | PLCOca-  NHSco^b^ | NHSca-  NHSco^c^ | NHSca-  PLCOco^d^ |
| --- | --- | --- | --- | --- |
| 1^st^ PC | 1.602 (0.121) | 1.713 (0.135) | 1.970 (0.161) | 1.844 (0.151) |
| 2^nd^ PCs | 1.545 (0.116) | 1.545 (0.115) | 1.394 (0.096) | 1.409 (0.098) |
| 3^rd^ PCs | 1.124 (0.064) | 1.114 (0.064) | 1.241 (0.078) | 1.153 (0.068) |
| 4^th^ PCs | 1.084 (0.059) | 1.135 (0.064) | 1.106 (0.063) | 1.079 (0.060) |
| 5^th^ PCs | 1.038 (0.054) | 1.054 (0.056) | 1.037 (0.055) | 1.036 (0.054) |
| 6^th^ PCs | 1.045 (0.057) | 1.029 (0.052) | 1.048 (0.056) | 1.043 (0.055) |
| 7^th^ PCs | 1.038 (0.056) | 1.050 (0.055) | 1.034 (0.055) | 1.033 (0.054) |
| 8^th^ PCs | 1.027 (0.053) | 1.038 (0.054) | 1.031 (0.053) | 1.040 (0.055) |
| 9^th^ PCs | 1.038 (0.053) | 1.051 (0.055) | 1.047 (0.056) | 1.028 (0.054) |
| 10^th^ PCs | 1.049 (0.056) | 1.029 (0.054) | 1.037 (0.055) | 1.038 (0.055) |

Note: The over-dispersion factor (and the empirical type I error under the significant level of 0.05) are estimated by applying the 1-df Wald test statistic without the PC adjustment on the set of 241,238 genomic control SNPs.

^a^PLCOca-PLCOco is the original PLCO prostate cancer study.

^b^PLCOca-NHSco is the reconstructed study with prostate cancer cases from the PLCO, and external controls from NHS.

^c^NHSca-NHSco is the original NHS breast cancer study.

^d^NHSca-PLCOco is the reconstructed study with breast cancer cases from the NHS, and external controls from PLCO.
