# Supplementary material for: Population Substructure and Control Selection in Genome-Wide Association Studies
Source: PLoS One. 2008 Jul 2;3(7):e2551. doi: 10.1371/journal.pone.0002551 (PMC2432498; doi:10.1371/journal.pone.0002551)
Supplement: Table S5 — Results on the PLCOca-NHSco using the set of 12,536 SNPs on chromosome 20 in the principal components analysis (0.03 MB DOC) [file pone.0002551.s006.doc]

**Table S5. Results on the PLCOca-NHSco using the set of 12,536 SNPs on chromosome 20 in the principal components analysis**

| PCs chosen for | Over- | Significant level | | |
| --- | --- | --- | --- | --- |
| the adjustment | dispersion | 0.05 | 0.01 | 0.001 |
| 0 PC | 1.076 | 0.0592 | 0.0131 | 0.0016 |
| 1^st^ PC | 1.066 | 0.0579 | 0.0126 | 0.0014 |
| 1-2 PCs | 1.064 | 0.0577 | 0.0126 | 0.0014 |
| 1-3 PCs | 1.065 | 0.0577 | 0.0126 | 0.0014 |
| 1-4 PCs | 1.067 | 0.0581 | 0.0127 | 0.0015 |
| 1-5 PCs | 1.067 | 0.0580 | 0.0126 | 0.0015 |
| 1-6 PCs | 1.066 | 0.0580 | 0.0127 | 0.0015 |
| 1-7 PCs | 1.058 | 0.0570 | 0.0123 | 0.0014 |
| 1-8 PCs | 1.057 | 0.0567 | 0.0122 | 0.0014 |
| 1-9 PCs | 1.057 | 0.0568 | 0.0123 | 0.0014 |
| 1-10 PCs | 1.056 | 0.0569 | 0.0122 | 0.0014 |

Note: The over-dispersion factor and empirical type I errors under various significant levels are estimated by applying the 1-df Wald test statistic with the adjustment of selected PCs on the all autosomal SNPs except those on chromosome 20.
