# Supplementary figures and images for: Population Substructure and Control Selection in Genome-Wide Association Studies
Source: PLoS One. 2008 Jul 2;3(7):e2551. doi: 10.1371/journal.pone.0002551 (PMC2432498; doi:10.1371/journal.pone.0002551)

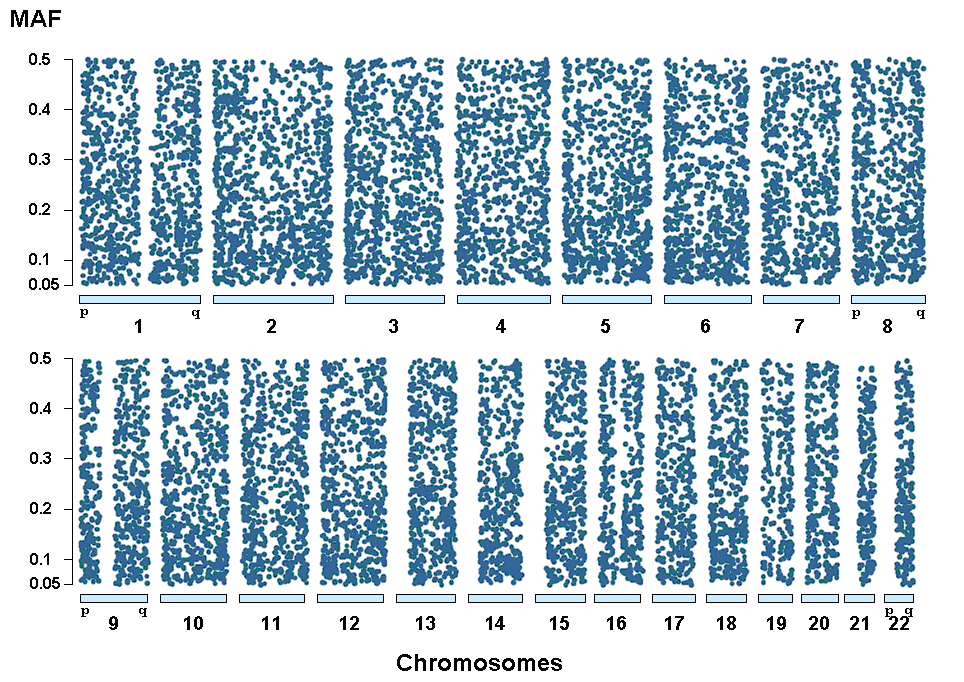

Supplement: Figure S1 — Distribution of the minor allele frequency of 12,898 population substructure inference SNPs on each autosome. Each SNP's physical map position on the chromosome is based on the reference genome build 36.2 and the latest dbSNP build 128. One SNP (rs3789771) has no map information and is excluded from the figure. (0.22 MB TIF) [file pone.0002551.s007.tif]

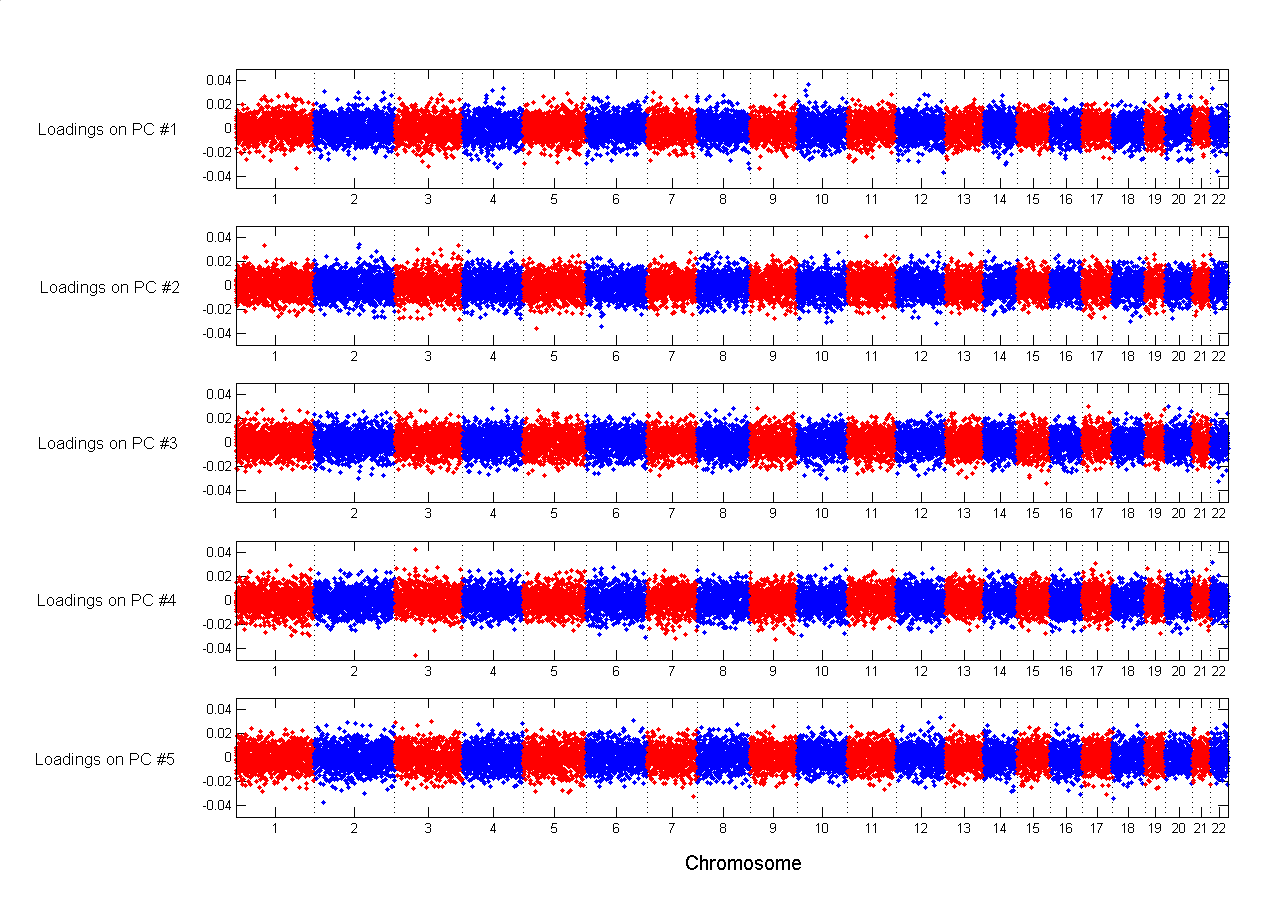

Supplement: Figure S2 — SNP Loadings from PCA using 12,898 population substructure inference SNPs in the PLCO prostate cancer study. SNPs are organized according to the order of their chromosome positions. (0.12 MB TIF) [file pone.0002551.s008.tif]

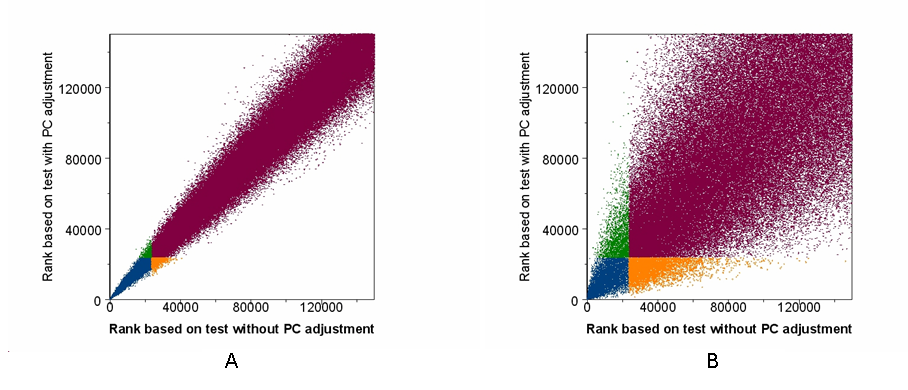

Supplement: Figure S3 — SNP ranking correlation in breast cancer studies. In each plot, SNPs' rankings based on the 1 d.f. Wald test on 475,116 testing autosomal SNPs without PC adjustment are compared with their rankings based on the 1 d.f. Wald test with adjustment for PCs chosen by the permutation procedure. The SNPs in blue are ranked among the top 5% by tests both with and without PC adjustment. The SNPs in green and orange are ranked among the top 5% by only one of the tests. A) Results based on the original breast cancer study (breast cancer cases and controls from NHS). The 2nd PC was chosen for PS correction. B) Results based on the reconstructed breast cancer study using external controls (breast cancer cases from NHS, and external controls from PLCO). The 1st, 2nd and 3rd PCs were chosen for PS correction. (0.29 MB TIF) [file pone.0002551.s009.tif]

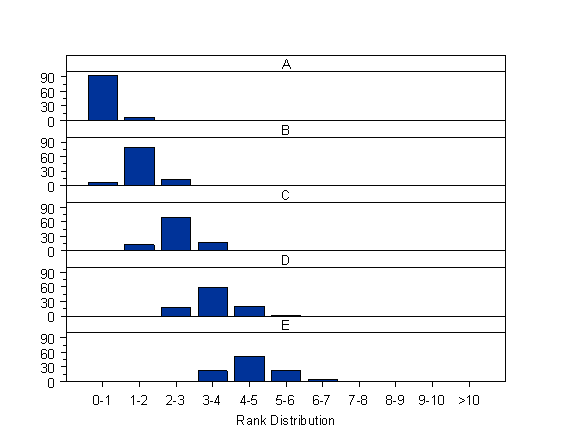

Supplement: Figure S4 — The conditional ranking distribution for the original NHS breast cancer study. Each plot shows the histogram of ranks according to the test without PC adjustment for SNPs ranked within a given range by the test with the adjustment for the 2nd PC (chosen by the proposed permutation procedure). The ranking ranges (%) are shown on the horizontal axis. The frequencies (%) are shown on the vertical axis. A) The histogram of ranks for SNPs ranked in the top 0–1% by the test with PC adjustment. B) The histogram of ranks for SNPs ranked in the top 1–2% by the test with PC adjustment. C) The histogram of ranks for SNPs ranked in the top 2–3% by the test with PC adjustment. D) The histogram of ranks for SNPs ranked in the top 3–4% by the test with PC adjustment. E) The histogram of ranks for SNPs ranked in the top 4–5% by the test with PC adjustment. (0.02 MB TIF) [file pone.0002551.s010.tif]

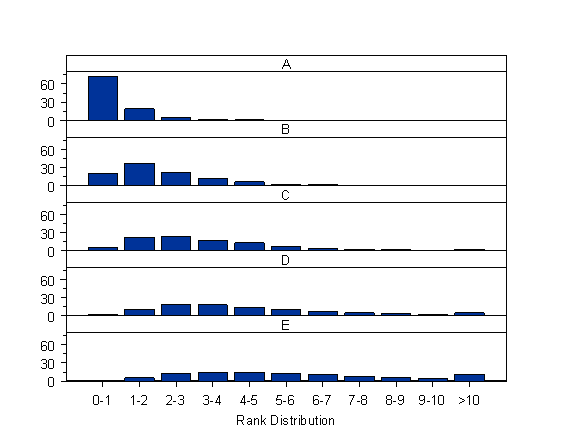

Supplement: Figure S5 — The conditional ranking distribution for the reconstructed breast cancer study using external controls. Each plot shows the histogram of ranks according to the test without PC adjustment for SNPs ranked within a given range by the test with the adjustment for the 1st, 2nd, and 3rd PCs (chosen by the proposed permutation procedure). The ranking ranges (%) are shown on the horizontal axis. The frequencies (%) are shown on the vertical axis. A) The histogram of ranks for SNPs ranked in the top 0–1% by the test with PC adjustment. B) The histogram of ranks for SNPs ranked in the top 1–2% by the test with PC adjustment. C) The histogram of ranks for SNPs ranked in the top 2–3% by the test with PC adjustment. D) The histogram of ranks for SNPs ranked in the top 3–4% by the test with PC adjustment. E) The histogram of ranks for SNPs ranked in the top 4–5% by the test with PC adjustment. (0.02 MB TIF) [file pone.0002551.s011.tif]

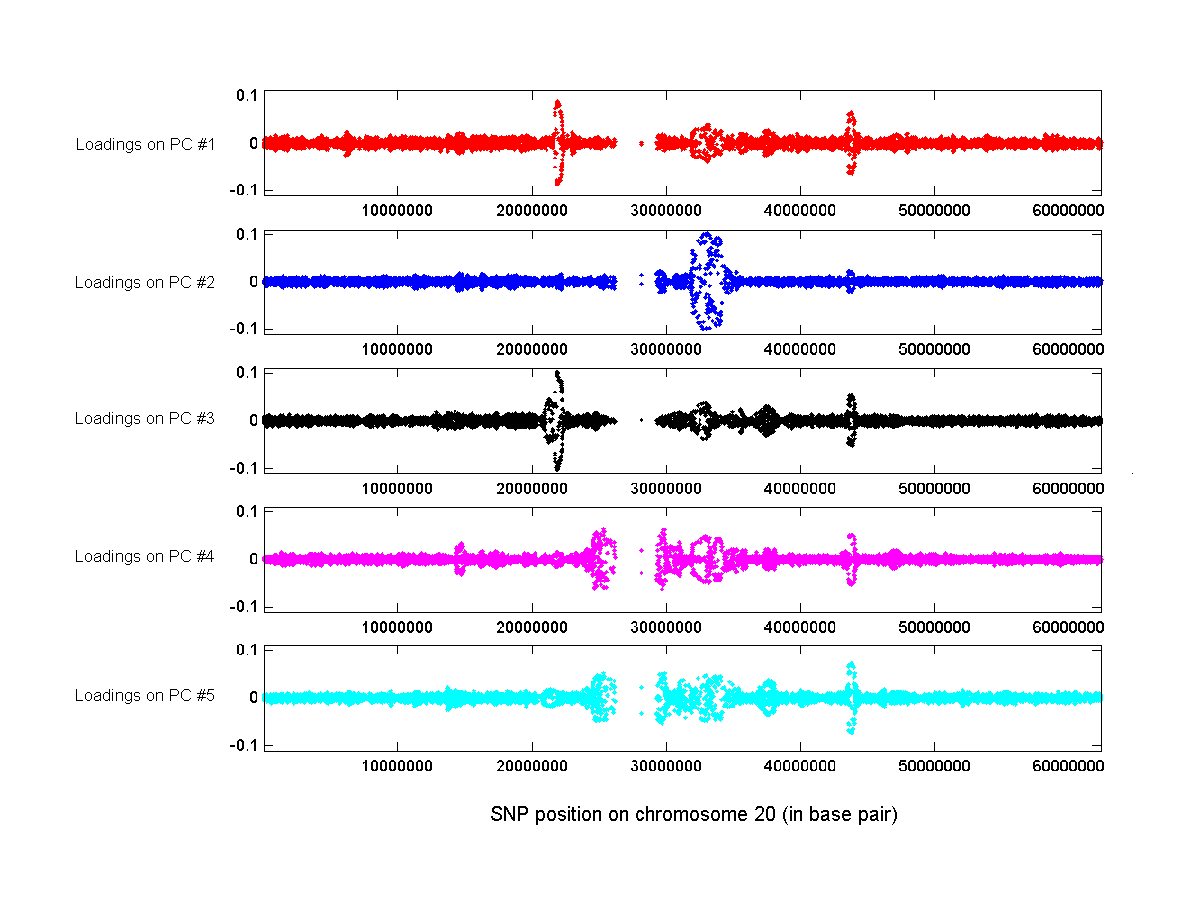

Supplement: Figure S6 — SNP loadings from PCA using SNPs on chromosome 20 in prostate cancer study using external controls. SNPs are organized according to their positions (in base pair) on chromosome 20. (0.09 MB TIF) [file pone.0002551.s012.tif]
